# Dietary Iron Restriction Improves Muscle Function, Dyslipidemia, and Decreased Muscle Oxidative Stress in Streptozotocin-Induced Diabetic Rats

**DOI:** 10.3390/antiox11040731

**Published:** 2022-04-07

**Authors:** Manuel Alejandro Vargas-Vargas, Alfredo Saavedra-Molina, Mariana Gómez-Barroso, Donovan Peña-Montes, Christian Cortés-Rojo, Huerta Miguel, Xochitl Trujillo, Rocío Montoya-Pérez

**Affiliations:** 1 Instituto de Investigaciones Químico-Biológicas, Universidad Michoacana de San Nicolás de Hidalgo, Francisco J. Múgica S/N, Col. Felicitas del Río, Morelia 58030, Mexico; 1371614e@umich.mx (M.A.V.-V.); francisco.saavedra@umich.mx (A.S.-M.); 0939531k@umich.mx (M.G.-B.); 0618853j@umich.mx (D.P.-M.); christian.cortes@umich.mx (C.C.-R.); 2 Centro Universitario de Investigaciones Biomédicas, Universidad de Colima, Av. 25 de Julio 965, Las Víboras, Colima 24040, Mexico; huertam@ucol.mx (H.M.); rosio@ucol.mx (X.T.)

**Keywords:** diabetes, fatigue, iron, oxidative stress

## Abstract

Diabetes mellitus is a chronic degenerative disease characterized by hyperglycemia and oxidative stress. Iron catalyzes free radical overproduction. High iron concentrations have previously been reported to promote an increase in oxidative stress; however, the effect of iron restriction in diabetes has not yet been explored, so we tested to see if iron restriction in diabetic rats reduces oxidative damage and improved muscle function. Wistar rats were assigned to 4 groups: Control; Diabetic; Diabetic rats with a high iron diet, and Diabetic with dietary iron restriction. After 8 weeks the rats were sacrificed, the muscles were extracted to prepare homogenates, and serum was obtained for biochemical measurements. Low iron diabetic rats showed an increase in the development of muscle strength in both muscles. Dietary iron restriction decreased triglyceride concentrations compared to the untreated diabetic rats and the levels of extremely low-density lipoproteins. Aggravation of lipid peroxidation was observed in the diabetic group with a high iron diet, while these levels remained low with iron restriction. Iron restriction improved muscle strength development and reduced fatigue times; this was related to better lipid profile control and decreased oxidant stress markers.

## 1. Introduction

Diabetes mellitus is characterized by metabolic dysfunction resulting from changes in insulin secretion and/or resistance to its actions [1]. Chronic hyperglycemia in diabetes increases oxidative stress, causing cell damage that eventually leads to acute and chronic complications [2,3,4]. Muscle fatigue is an acute symptom of diabetes, causing loss of strength and mass in muscle [5]. Oxidative stress is a key factor involved in the development of muscle fatigue [6,7]. Dyslipidemia is another hallmark of diabetes associated with a higher risk of cardiovascular disease [8]; hereby, the decrease in blood lipids improves cardiovascular outcomes [9,10]. Dyslipidemia has been related to impaired muscle function in rodents [11] and humans [12]. Thus, dyslipidemia may enhance the deleterious effects of oxidative stress on muscles.

On the other hand, high intracellular iron concentrations (i.e., iron overload) enhance oxidative stress by catalyzing ROS production via the iron/H_2_O_2_-based formation of hydroxyl radicals [13]. Oxidative stress due to iron overload accelerates both the development and progression of diabetic complications [14,15]. Accordingly, the role of iron overload in the impaired metabolism of lipids and carbohydrates has been confirmed in diabetic rats by improving dyslipidemia and glucose homeostasis via dietary iron restriction [16]. However, it is unknown whether dietary iron restriction also improves muscle performance in diabetes. Thus we aimed to test if, besides alleviating dyslipidemia, iron restriction also improves muscle performance and oxidative stress in diabetic rats. Moreover, we fed rats with a high iron diet to induce iron overload and test if this phenomenon enhances the alterations elicited by diabetes in muscle function, blood lipids, and oxidative stress. 

## 2. Materials and Methods

### 2.1. Animals and Dietary Treatments

Male Wistar rats (weight, 200–250 g) were used and preserved under standard laboratory conditions with *ad libitum* food and water. All animal procedures were conducted following the Technical specifications for the production, use, care, and handling of laboratory animals (NOM-062-ZOO-1999, Ministry of Agriculture, Mexico City, México) and were approved by the Institutional Committee for the Use and Care of Animals of the Instituto de Investigaciones Químico-Biológicas of the Universidad Michoacana de San Nicolás de Hidalgo (UMSNH) (Number 2018-06; April 2018). The standard control diet (D12050203) contained iron at 100 mg/kg (Research Diets, Inc., New Brunswick, NJ, USA). The low iron diet (D03072501, Research Diets, Inc.) had 3 mg iron/kg. The high iron diet consisted of a standard control diet plus oral administration of polymaltosed iron (Takeda, Naucalpan, México, S.A. de C.V., under license of Vifor International, Inc., St. Gallen, Switzerland) through an orogastric tube at a dose of 3 mg of iron/kg body weight per day, for 8 weeks, to establish a pattern of iron overload [16].

### 2.2. Induction of Diabetes and Experimental Design 

Experimental diabetes was induced by a single injection of streptozotocin (STZ) (Sigma-Aldrich, St. Louis, MO, USA) at a dose of 45 mg/kg body weight. STZ was dissolved in 0.1 M sodium citrate buffer (pH 4.5), and control rats were injected only with sodium citrate buffer. Blood glucose was measured two days after STZ injection, and rats with glucose levels >200 mg/dL were considered diabetic. Twenty four rats were divided into four groups of six animals each: (1) Control group (Control): fed only with standard rodent show diet; (2) Diabetic group (DB): diabetic rats fed only with standard rodent chow diet; (3) Diabetic plus high iron diet group (DB + IO): Diabetic rats with iron overload diet; (4) Diabetic rats with iron restriction group (DB + IR). Fresh blood samples were collected from the rat tail vein, and glucose levels were determined using a glucometer (ACCU-CHEK^®^ Active, Roche Diagnostics, Mannheim, Germany) every week during the experimental stage.

### 2.3. Muscle Extraction and Tissue Preparation

Animals were sacrificed by decapitation once the experimental period was over. The soleus and extensor digitorum longus (EDL) muscles of one limb were extracted and placed on a Petri dish with Krebs-Ringer buffer (75 mM KCl, 118 mM NaCl, 1.18 mM MgSO_4_, 1.18 mM KH_2_PO_4_, 24.8 mM NaHCO_3_, 2.08 g/L glucose, pH 7.4) for muscle tension tests. The muscles of the other limb were homogenized using Krebs Ringer buffer to assess oxidative stress biomarkers (lipid peroxidation, ROS, and glutathione), the homogenates were prepared and were preserved at a temperature of −70 °C until their subsequent use. The protein content of muscle homogenates was determined using the Biuret procedure with slight modifications using bovine serum albumin protein as the standard [17].

### 2.4. Hemoglobin Measurement

Hemoglobin (Hb) content was determined using the method by Drabkin and Sahli [18]. 10 μL heparinized blood was added to 2.5 mL of reactive solution (0.012 mM K_3_ [Fe (CN)_6_], 1.54 mm KCN, and 0.04 mM K_2_HPO_4_) and incubated for 5 min at room temperature. The optical density was recorded at 540 nm on a Perkin-Elmer Lambda 18 UV/vis spectrophotometer. Hb content was expressed in mg/dL blood and was calculated using a calibration curve with BSA.

### 2.5. Force Development and Fatigue Induction

The soleus and EDL muscles obtained from one of the hind legs were used for tension records. Both muscles were positioned in a Petri dish with a resin bottom (Sylgard), fixed with entomological pins immersed in Krebs-Ringer solution, and supplied with 95% CO_2_ and 5% O_2_. Excess fat and connective tissue were removed. Once cleaned, the muscle was mounted in a chamber for recording isometric tension, with its proximal end attached to the bottom of the chamber and the distal end to the hook of an optical transducer (World Precision Instruments, Sarasosa, FL, USA), which was connected to an amplifier (World Precision Instruments) and an analogic-to-digital interface (World Precision Instruments) for the acquisition of muscle tension MDAC software (World Precision Instruments) was used. Inside the recording chamber, two platinum electrodes were placed, linked to a stimulation isolating unit (Grass) to carry out the fatigue protocol. This consisted of 300 ms pulses of 100 V and 50 Hz for EDL muscle and 45 Hz for soleus muscle. Stimulation was stopped after fatigue appeared (70% of force reduction). 

### 2.6. Lipid Profile

Serum was collected by centrifugation of blood samples at 3000× *g* for 5 min (Thermo Scientific Sorvall RC 6+ centrifuge). Next, a Fujifilm NX500i (Tokyo, Japan) system was used to analyze the total serum cholesterol (TC), triglyceride (TG), and high-density lipoprotein (HDL) using Spinreact (Girona, Spain) reagents following the supplier’s instructions. Very low-density lipoprotein (VLDL) was calculated using the following formula: VLDL = TG/5 [19].

### 2.7. Determination of Lipid Peroxidation

The thiobarbituric acid (TBA) method was used to determine lipid peroxidation in muscle homogenates [20]. 0.5 mg protein was resuspended in phosphate buffer (100 mM, pH 7.4), combined with a reagent solution (0.375% thiobarbituric acid (TBA), 15% trichloroacetic acid (TCA), and 0.25 M HCl). This mixture was vortexed and incubated in a boiling water bath for 25 min. The absorbance of the supernatant was measured at 532 nm using a Shimadzu UV-2550 spectrophotometer. The results were expressed as thiobarbituric acid reactive substances (TBARS) per milligram of protein.

### 2.8. ROS Measurements

The cell-permeable fluorescent probe 2′,7′-dichlorodihydrofluorescein diacetate (H_2_DCFDA) determined ROS levels. 0.5 mg muscle protein was resuspended in 2 mL of buffer with 100 mM KCl, 10 mM 4-(2-hydroxyethyl)-1-piperazineethanesulfonic acid (HEPES), 3 mM KH_2_PO_4_ and 3 mM MgCl_2_ (pH 7.4) were incubated with 5 μL of 2′,7′-dichlorofluorescein diacetate (H_2_DCFDA) during 15 min at 4 °C under constant stirring. Fluorescence changes were detected in an RF-5301PC spectrofluorometer (Shimadzu Corporation, Kyoto, Japan) (λ_ex_ 485 nm; λ_em_ 520 nm) [21].

### 2.9. Measurement of Glutathione

Glutathione redox status was determined with the method by Rahman [22] with slight modifications. Total glutathione (GSH + GSSG) content was determined in 0.5 mg protein. Samples were suspended in 0.6% sulfosalicylic acid and 0.1% Triton X-100 in a 0.1 M potassium phosphate buffer (pH 7.5). This mixture was sonicated three times during 5 s and placed on ice for 20 s between each sonication cycle, succeeded by two freezing/thawing cycles, and centrifuged at 6500× *g*. Then, the supernatant was mixed with a potassium phosphate buffer with 0.1 units/mL glutathione reductase and 100 µM 5,5′-Dithiobis (2-nitrobenzoic acid) (DTNB). The reaction was started by adding 50 µM β-NADPH and monitored for 5 min at 412 nm in a UV/vis spectrophotometer (Shimadzu UV-2550, Kyoto, Japan). For determinations of oxidized glutathione (GSSG), samples were incubated for 1 h at room temperature with 0.2% 4-vinylpyridine to derivatize reduced glutathione (GSH). Then, the samples were treated as described above for determinations of GSH + GSSG. GSH concentration was calculated by subtracting the concentration of GSSG from the concentration of GSH + GSSG.

### 2.10. Statistical Analysis

All the data are expressed as the mean ± standard error (SEM) of at least *n* = 6. Statistical significance was evaluated using the Student *t*-test or ANOVA followed by a post hoc Tukey test; statistical significance was set at *p* < 0.05. The analyses were done with GraphPad Prism 6 (Prism 6.0, GraphPad Software Inc., San Diego, CA, USA). 

## 3. Results

### 3.1. Effect of Dietary Iron on Blood Glucose and Body Weight in STZ-Diabetic Induced Rats 

**Figure 1:**
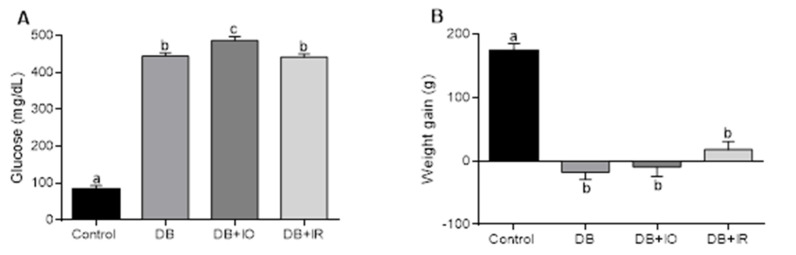
Fasting serum glucose (**A**) and Weight gain (**B**). Weight gain was determined by subtracting the animals’ weight after eight-week treatments with the diets, determined right before sacrifice, minus the weight at the beginning of the treatments. Data are expressed as the mean ± standard error. *p* < 0.05 (*n* = 6, ANOVA plus Tukey’s post hoc test). DB + IO, diabetic rats with iron overload; DB + IR, diabetic rats with iron—restricted diet. Different letters (a–c) represent statistically significant differences.

Glucose levels in all the diabetic groups (i.e., DB, DB + IO, DB + IR) remained several-fold higher than in the control group (Figure 1A). Regarding weight gain at the end of the experimental period (Figure 1B), the control group showed a weight gain of 158 g, while diabetic rats exhibited a negative balance in weight gain prevented in the DB + IR group, although weight gain was not as high as in the control group. Nevertheless, this effect was not statistically significant.

### 3.2. Effects of Dietary Iron on Hemoglobin

**Figure 2:**
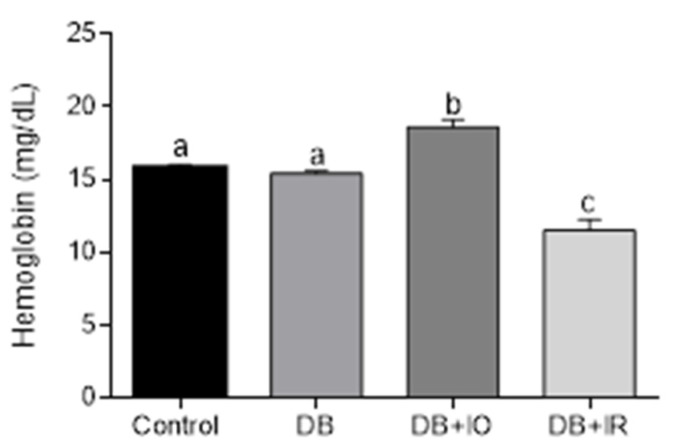
Effect of iron on hemoglobin levels. Data are presented as group mean ± standard error. *p* < 0.05 (*n* = 6, ANOVA plus Tukey’s post hoc test). Different letters represent statistically significant differences. DB + IO, diabetic rats with iron overload; DB + IR, diabetic rats with iron restriction diet.

Hemoglobin content is shown in Figure 2. Higher hemoglobin levels were found in the DB + IO, while the DB + IR group exhibited lower levels, even below the levels in the control group. It should be stressed that animals from the DB + IR group did not develop anemia, as hemoglobin levels were above 10 mg/dL.

### 3.3. Effects of Dietary Iron on Muscle Strength Development and Fatigue

**Figure 3:**
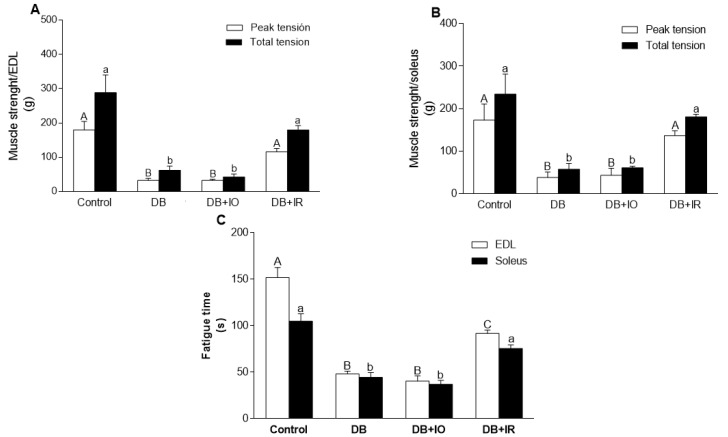
Effect of iron on muscle strength development in EDL (**A**), soleus muscle (**B**) peak tension (open bars) and solid bars (total tension). Effect of iron on fatigue (**C**) in EDL (open bars) and soleus muscles (solid bars). Data are presented as group mean ± standard error. *p* < 0.05 (*n* = 6, ANOVA plus Tukey’s post hoc test). (**A**–**C**) represent the statistically significant differences between the different groups of the peak tension (open bars); a, b represent the statistically significant difference between the different groups of the total tension (solid bars); DB + IO, diabetic rats with iron overload; DB + IR, diabetic rats with iron restriction diet.

The effects of dietary iron on muscle function are depicted in Figure 3. The DB and DB + IO groups displayed a similar degree of strength loss in both EDL (Figure 3A) and soleus (Figure 3B) muscles. The iron restriction group shows an increase in the development of muscular strength in both muscles. Moreover, the time for the development of muscle fatigue was shorter in both the DB and DB + IO groups (Figure 3C). These negative effects were counteracted by iron restriction in the DB + IR group, although the fatigue time did not reach the levels of the control group.

### 3.4. Effects of Iron on Serum Lipid Profile

**Figure 4:**
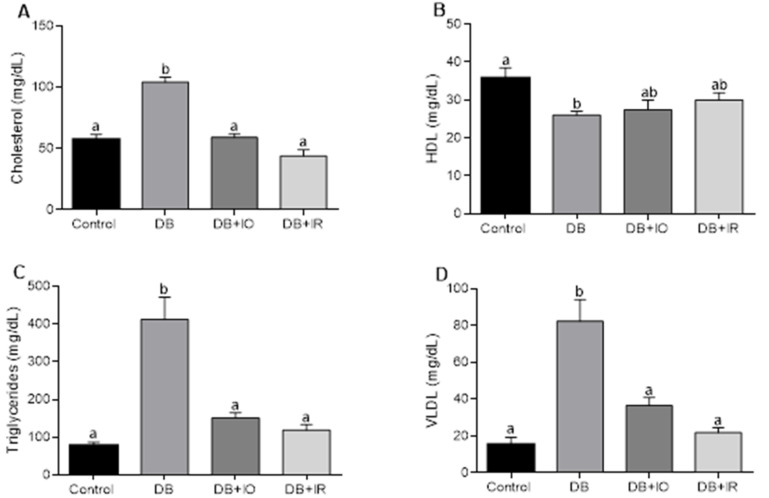
Serum levels of cholesterol (**A**), high-density lipoproteins (HDL) (**B**), triglycerides (**C**), and very-low-density lipoproteins (VLDL) (**D**). Data are presented as group mean ± standard error. *p* < 0.05 (*n* = 6, ANOVA plus Tukey’s post hoc test). Different letters represent statistically significant differences. DB + IO, diabetic rats with iron overload; DB + IR, diabetic rats with iron restriction diet.

Serum cholesterol levels are shown in Figure 4A. Compared to the control group, cholesterol levels were twofold higher in the DB group, while no significant changes were observed in the DB + IO and DB + IR groups. No differences between the groups were detected in serum HDL levels (Figure 4B). Serum triglycerides levels are shown in Figure 4C. Compared to the control group, triglycerides dramatically increased in the DB group. This was fully counteracted in the DB + IO and DB + IR groups, and similar behavior was observed for VLDL levels (Figure 4D).

### 3.5. Effect of Iron on Oxidative Stress Markers

**Figure 5:**
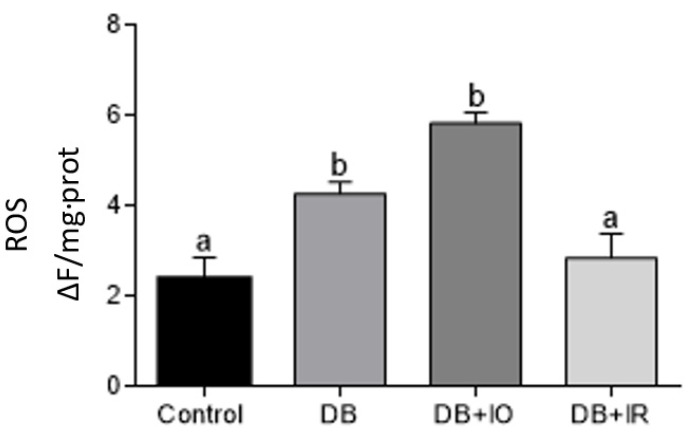
Effect of iron on the levels of ROS in muscle homogenates from control rats. Data are presented as group mean ± standard error. *p* < 0.05 (*n* = 6, ANOVA plus Tukey’s post hoc test). Different letters represent statistically significant differences. DB + IO, diabetic rats with iron overload; DB + IR, diabetic rats with iron restriction diet.

Muscle ROS levels are depicted in Figure 5. ROS levels doubled in the DB group compared to the control group, and they increased almost thrice in the DB + IO group. In contrast, dietary iron restriction decreased ROS levels in the DB + IR group almost to control levels (Figure 5).

**Figure 6:**
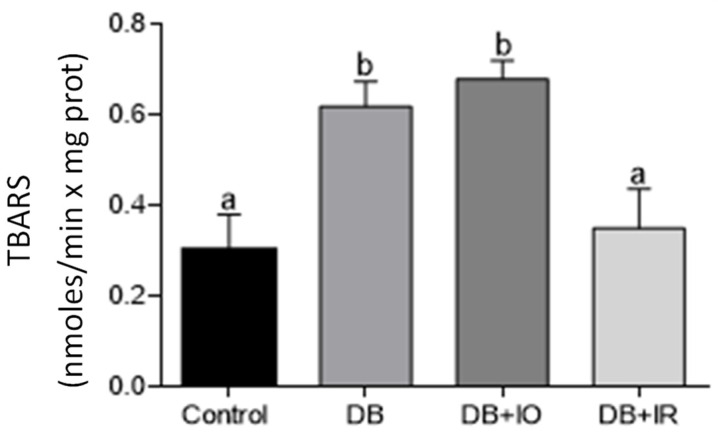
Effects of iron on muscle lipid peroxidation. Data are presented as group mean ± standard error. *p* < 0.05 (*n* = 6, ANOVA plus Tukey’s post hoc test). Different letters indicate statistically significant differences. DB + IO, diabetic rats with iron overload; DB + IR, diabetic rats with iron restriction diet.

A similar trend was observed in lipid peroxidation (Figure 6), as the levels of this parameter doubled in both the DB and DB + IO groups and decreased to the levels of the control group in the DB + IR group.

**Figure 7:**
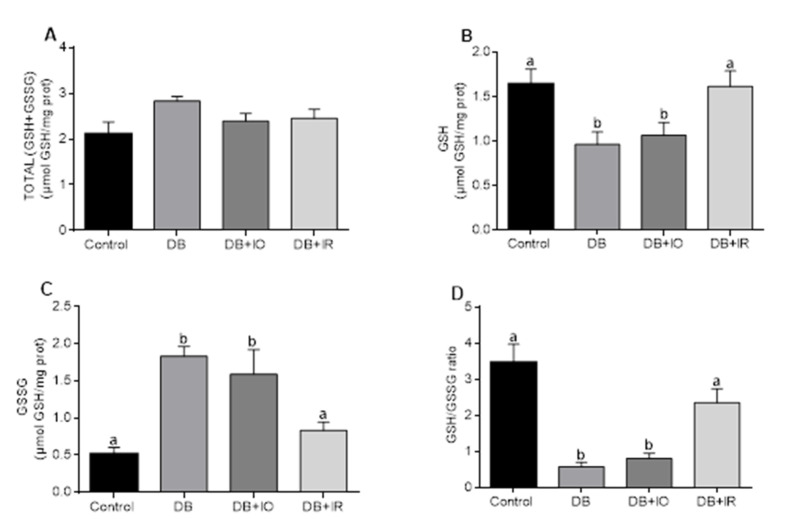
Effects of dietary iron on total glutathione (**A**), reduced glutathione (GSH) (**B**), oxidized glutathione (GSSG) (**C**), and GSH/GSSG ratio (**D**). Data are presented as group mean ± standard error. *p* < 0.05 (*n* = 6, ANOVA plus Tukey’s post hoc test). Different letters (a,b) indicate statistically significant differences. DB + IO, diabetic rats with iron overload; DB + IR, diabetic rats with iron restriction diet.

The glutathione redox status in the muscle is shown in Figure 7. No differences in total glutathione levels were observed between the different experimental groups (Figure 7A). GSH levels decreased in both the DB and DB + IO groups compared to the control group (Figure 7B). On the contrary, GSSG levels were higher in both the DB and DB + IO groups than in the control group (Figure 7C). These changes in glutathione redox state render a severe state of oxidative stress in the muscle of both the DB and DB + IO groups, as the GSH/GSSG ratio was considerably lower in these groups compared to the control group (Figure 7D). In contrast, dietary iron restriction almost normalized in the DB + IR group all the alterations in the levels of GSH (Figure 7B), GSSG (Figure 7C), and GSH/GSSG ratio (Figure 7D).

## 4. Discussion

Dietary iron restriction improved skeletal muscle function in type 1 STZ-induced diabetic rats, accompanied by decreased muscle oxidative stress and improved dyslipidemia. Of note, the protocol of iron restriction used in this study did not induce anemia, as this condition occurs in rats when hemoglobin is lower than 10 mg hemoglobin/dL.

No weight gain was observed in the diabetic rats throughout the experimental period, consistent with the same phenotype seen in previous studies [23]. Iron restriction improves insulin sensitivity in obesity, leading to improved glucose levels [24]. STZ induces diabetes by depleting insulin production due to pancreatic β-cell destruction [25] rather than by impairing insulin sensitivity; this may explain the lack of a hypoglycemic effect of iron deprivation in this model of diabetes.

The more deleterious effects on muscular strength were observed in both the DB and DB + IO groups. This may be related to the fatigue commonly described in diabetic individuals [6], as iron overload has been observed in muscles with insulin resistance [26]. Moreover, it has also been described that iron overload leads to the development of fatigue [27] and negatively affects muscle contractility [28]. Thus, the maximum negative effect on fatigue is caused by diabetes itself, without iron overload worsening this outcome. Iron plays an essential role in the development of diabetes, and it promotes oxidative stress [15], which might be involved in the impaired ability to develop muscle strength [29]. Conversely, the iron restriction positively affected muscle strength development, both in the soleus muscle and in the EDL muscle. Little is known about the direct impact that iron has on the development of muscle strength in diabetes; however, it has been determined that iron restriction causes positive effects in various pathologies such as obesity [24].

The positive effects of iron restriction on dyslipidemia (Figure 4) agree with the beneficial effects of this approach in altered lipid and glucose metabolism in a type 2 diabetes model. It was also found that iron restriction decreased systemic oxidative stress [30].

Unexpectedly, iron overload also counteracted dyslipidemia in the DB + IR group. Iron overload has been reported to increase blood triglycerides in non-diabetic rats by enhancing hepatic oxidative stress, impairing the expression of peroxisome proliferator-activated receptor-alpha, and decreasing fatty acid beta-oxidation. Furthermore, iron overload promotes hepatic lipid secretion by increasing the mRNA expression of apoB-100, which is associated with the assembly of VLDL, and by improving the assembly of apoB-100 into VLDL by increasing the expression of microsomal triglyceride transfer protein [31]. On the other hand, iron overload induces hypercholesterolemia by enhancing the mRNA expression of hydroxymetylglutaryl CoA reductase (HMG CoA) and the activity of acyl-CoA cholesterol acyltransferase (ACAT), thus increasing cholesterol synthesis and VLDL—cholesterol secretion [32,33]. We have no data to explain why iron overload had a hypoglycemia effect in STZ—induced diabetic rats. We can speculate that this resulted from impaired hepatic lipid synthesis elicited by the combined effect of iron accumulation in the liver [34] and the damage induced by diabetes in organelles closely related to lipid metabolism like mitochondria [23], leading to a catastrophic bioenergetic collapse decreasing ATP synthesis. This, in turn, may impair biosynthetic pathways requiring ATP, such as cholesterol synthesis or biogenesis of VLDL transport vesicles [35], which may have also contributed to generalized oxidative damage in biomolecules and cellular structures [34,36,37]. Therefore, decreased dyslipidemia would result from the harmful effects of iron on bioenergetics and cellular redox state rather than an adaptive response to enhanced iron concentrations and oxidative stress.

The diabetic rats exhibited higher VLDL levels, which are closely associated with coronary artery calcification in diabetes, and this association becomes more substantial when triglyceride levels are above normal levels [38]. Individuals with type 2 diabetes frequently exhibit macrovascular complications of atherosclerotic cardiovascular disease. It has been shown that HDL is protective against atherosclerosis [39]. The decrease in HDL observed in the diabetic rats and the null effect of iron restriction on impaired HDL levels (Figure 4B) suggests that the beneficial effects of iron restriction might not be enough to counteract atherosclerotic disease in the microvasculature, as HDL may attenuate inflammation by decreasing the activation of mediators of this process such as the nuclear factor kappa B (NF-κB), which, in turn, suppresses the expression of HIF-1α, VEGFA, and VEGFR2 [40].

There is a positive correlation between the amount of intake of iron and ROS levels in skeletal muscle (Figure 5). In agreement, decreased ROS levels were accompanied by lower oxidative stress, as supported by the higher GSH/GSSG ratio observed with iron restriction in the DB + IR group (Figure 7). Diabetes set higher levels of oxidative stress even in the absence of excessive iron intake, as oxidative stress markers were not higher in the DB + IO group than in the DB group. On the other hand, iron restriction improved muscle strength and significantly delayed the onset of fatigue (Figure 3). In this regard, it is known that increased levels of ROS have many deleterious effects on skeletal muscle, such as reducing force generation and atrophy [30], which is consistent with the data in Figure 3 and Figure 4. Our findings show that iron restriction also lowers lipid peroxidation and improves glutathione redox status (Figure 6 and Figure 7D), suggesting that iron restriction in the diet decreases radical hydroxyl production, reducing the damage to membrane lipids and preserving the glutathione redox status.

## 5. Conclusions

The addition of iron in the diet does not exacerbate the damage caused by diabetes. However, the restriction of iron promotes positive effects. It enhances muscle strength, delays fatigue, improves lipid homeostasis in diabetes, and also decreases oxidative stress markers such as ROS levels and TBARS levels, and decreases the GSH/GSSG ratio.

## Data Availability

Data is contained within the article.
